# Statistical actuarial estimation of the Capitation Payment Unit from copula functions and *deep learning*: historical comparability analysis for the Colombian health system, 2015–2021

**DOI:** 10.1186/s13561-022-00416-5

**Published:** 2023-02-24

**Authors:** Oscar Espinosa, Valeria Bejarano, Jeferson Ramos, Boris Martínez

**Affiliations:** 1 Economic Models and Quantitative Methods Research Group, Centro de Investigaciones para el Desarrollo, Universidad Nacional de Colombia, Bogotá, D.C., Colombia; 2 Department of Mathematics, Universidad Nacional de Colombia, Bogotá, D.C., Colombia

**Keywords:** Pure risk premium, Health system, Copulas, Artificial neural networks, Actuarial science, C45, C51, G22, I13

## Abstract

The Capitation Payment Unit (CPU) financing mechanism constitutes more than 70% of health spending in Colombia, with a budget allocation of close to 60 trillion Colombian pesos for the year 2022 (approximately 15.7 billion US dollars). This article estimates actuarially, using modern techniques, the CPU for the contributory regime of the General System of Social Security in Health in Colombia, and compares it with what is estimated by the Ministry of Health and Social Protection. Using freely available information systems, by means of statistical copulas functions and artificial neural networks, pure risk premiums are calculated between 2015 and 2021. The study concludes that the weights by risk category are systematically different, showing historical pure premiums surpluses in the group of 0–1 years and deficits (for the regions normal and cities) in the groups over 54 years of age.

## Introduction

The General System of Social Security in Health (*Sistema General de Seguridad Social en Salud*, SGSSS for its acronym in Spanish) of Colombia has different financing mechanisms for its operation; the most important in terms of monetary magnitudes is the so-called Capitation Payment Unit (CPU). This *‘health insurance premium’*, currently calculated by the Ministry of Health and Social Protection (MHSP), has, since 2006, been computed based on three variables (risk adjusters): age, sex and region [25].

The purpose of the CPU is to finance a set of health technologies (drugs, procedures, supplies, medical devices, etc.), known as the Health Benefits Plan (HBP-CPU), which configures a collective protection mechanism for the right to health under a mandatory insurance scheme [9].

For example, for the year 2020, the SGSSS Resources Administrator (ADRES for its acronym in Spanish), the entity in charge of making the recognition and payment of the CPU to health insurers (called Entities Administrators of Health Benefit Plans, EAHBP), made transfers of about 48.5 trillion Colombian pesos (COP) for the contributory (CR)^1^ and subsidized (SR)^2^ regimes, distributed in similar proportions [1].

In a context of budgetary restrictions – common to all countries, of any income level – and in the face of an evident growing demand for more and better health technologies for the inhabitants, the financial sustainability of the SGSSS must be ensured, maximizing as far as possible the results in terms of the health of the population of the entire national territory. The pressures of health spending derived from the extensions of the HBP-CPU are a constant challenge for health systems, therefore, the constant study of the sufficiency of the cost of health risk management should be a priority evaluation issue for the care of state finances.

In this scenario, the analysis of the CPU's pricing becomes relevant. In this regard, the specialized literature has investigated alternatives for risk adjustment in SGSSS health spending [4, 21, 43, 44]. However, no studies have been found that develop a particular method for calculating the CPU of the risk groups defined by the legislation. The only antecedents are the official documents of the MHSP and the investigation by Basto et al. [3], which focuses exclusively on SR.

Because of this, the present research aims to estimate actuarially the pure risk premiums for CR^3^ by means of copulas functions and *deep learning* approximations, and to compare the estimated monetary values with those defined by the resolutions, for the years 2015 to 2021. [27–30, 32, 34, 36]. This will allow reviewing and contrasting the budget allocations that have been made over time based on real-world evidence and taking note of possible improvements in the computation of the financial calculation of health risk management in Colombia.

From 2015 to date, the MHSP has estimated the pure risk premiums for 56 groups that categorize the population affiliated with the health system. For this reason, the analysis period starts from that year and the estimates are made using the same groups. The 56 groups consist of the combinations of the categories of the variables: i) region: normal, remote, cities and special and ii) age/sex group: less than 1 year, 1–4 years, 5–14 years, 15- 18 years (men), 15–18 years (women), 19–44 years (men), 19–44 years (women), 45–49 years, 50–54 years, 55–59 years, 60–64 years, 65–69 years, 70–74 years, and 75 years or more.

This paper is structured as follows. The first section presents the historical context for the CPU and its pricing in the SGSSS. The second section offers a descriptive analysis of the data of interest and the new methodological proposal for estimating the statistical-actuarial pricing models. The third section presents the most relevant results and findings on the actuarial variables of frequency, severity and pure risk premiums. Finally, the fourth section contains the final considerations of the research, its main limitations and some proposals for future research on the subject.

## Historical context of the CR-CPU and its pricing

The social bodies responsible for establishing the values of the CPU have been in historical order: the National Council for Social Security in Health, the Health Regulation Commission, and (currently) the Directorate for the Regulation of Health Insurance Benefits, Costs and Rates of the MHSP. Since 2010, unlike previous years, the sufficiency studies use a clear actuarial concept, based on the fundamental insurance equation, assuming that the CPU can be understood as the division between the expected value of health costs and the population exposed to health risk [53].

From the statistical-actuarial approach, pricing methods are used to build premiums that cover the losses of the insured's subscribed risks, that is, that are sufficient, with a high degree of confidence [6, 12]. To estimate the CPU rate, the MHSP has used the method called the expected loss ratio, which is based on the quotient between the calculated loss ratio and the permissible loss ratio of the EAHBP (which according to Law 1438 of 2011 is of the order of 0.9 for CR). The result indicates what is the necessary increase of the CPU to guarantee the financial sufficiency of the SGSSS  [35].

In this context, the MHSP projects costs, income and those exposed to risk. For the first variable, it applies different trend adjustment factors to emulate future conditions: increases in the price level, frequency of claims, claims that are incurred but not reported (IBNR), HBP-CPU update, among others. For the second variable, it projects the possible items that make up the income of the EAHBP of the CR: income from CPU, copayments, moderating fees, recoveries from the Occupational Risk Administrators, income from registration and affiliation fees, income from the High-Cost Account, income Agreement 026 of 2012, as well as income from health promotion and prevention, among others. For the population exposed to risk, the MHSP makes adjustments for missing compensation and for the expected growth in the following year based on the population projections of the National Administrative Department of Statistics (*Departamento Administrativo Nacional de Estadística,* DANE for its acronym in Spanish).

The base information for the analysis of the regulatory entity refers to the calendar year immediately prior to the year of its realization, for example, the sufficiency study for the year 2019 estimates the increase in the CPU that will be sufficient during the year 2020 to finance health technologies, using real-world data from the year 2018. The latter are extracted, among other databases, from the reports on the provision of health services per affiliate issued by the EAHBP, the affiliate and compensation databases of the CR, the financial statements reported by the EAHBP to the entity for inspection, surveillance and control (National Health Superintendency) and the tariff manuals for health technologies financed by the CPU.

Now, for the case of this study, the conceptual approach considered to estimate the pure health risk premium is the product of frequency and severity, where the first factor corresponds to the ratio between the number of distinct people served and those exposed to health risk, 4 while the second factor is defined as the ratio between the total costs of health technologies over the number of distinct people served. Formally:

1 $$Pure\;health\;risk\;premium=\underbrace{Frequency}_\frac{Distinct\;people\;served}{Exposed\;to\;health\;risk}\ast\underbrace{Severity}_\frac{Total\;costs\;of\;health\;technologies}{Distinct\;people\;served}$$

This classic actuarial approach, unlike the expected loss ratio, allows the two variables of interest that describe the health risk to be modeled independently and specifically and to project a sufficient CR-CPU. The pure risk premium estimated in this way meets the theoretical properties desired in all premiums: additivity, independence, scale invariance, consistency and acceptability [55].

## Data and empirical strategy

### Data

For the statistical-actuarial estimation of the CR-CPU, it was necessary to have information on: i) those exposed to risk (equivalent population), from 2013 to 2020; ii) number of distinct people served by the SGSSS, from 2013 to 2019, and iii) severity (average costs) of health care, from 2013 to 2019. For the first variable, the Database of Affiliates (Base de Datos Única de Afiliados, BDUA for its acronym in Spanish) was used, which contains the information of the fully identified affiliates of the SGSSS who are covered by the HBP-CPU; for the second and third, Demand Management (Gestión de la Demanda, GD for its acronym in Spanish) was used through the Integrated Social Protection Information System (SISPRO for its acronym in Spanish), which includes all the expenses charged to the HBP-CPU by the EAHBP that exceed the validation meshes of the MHSP. GD can be considered a Sufficiency proxy, a confidential database and a fundamental input for the calculation of the CR-CPU from the regulatory entity.

Both BDUA and GD present disaggregations by sex, municipality code, department, among others, which allows the feasibility of this actuarial calculation, in accordance with the guidelines and risk adjusters pre-established in national legislation.

**Fig. 1:**
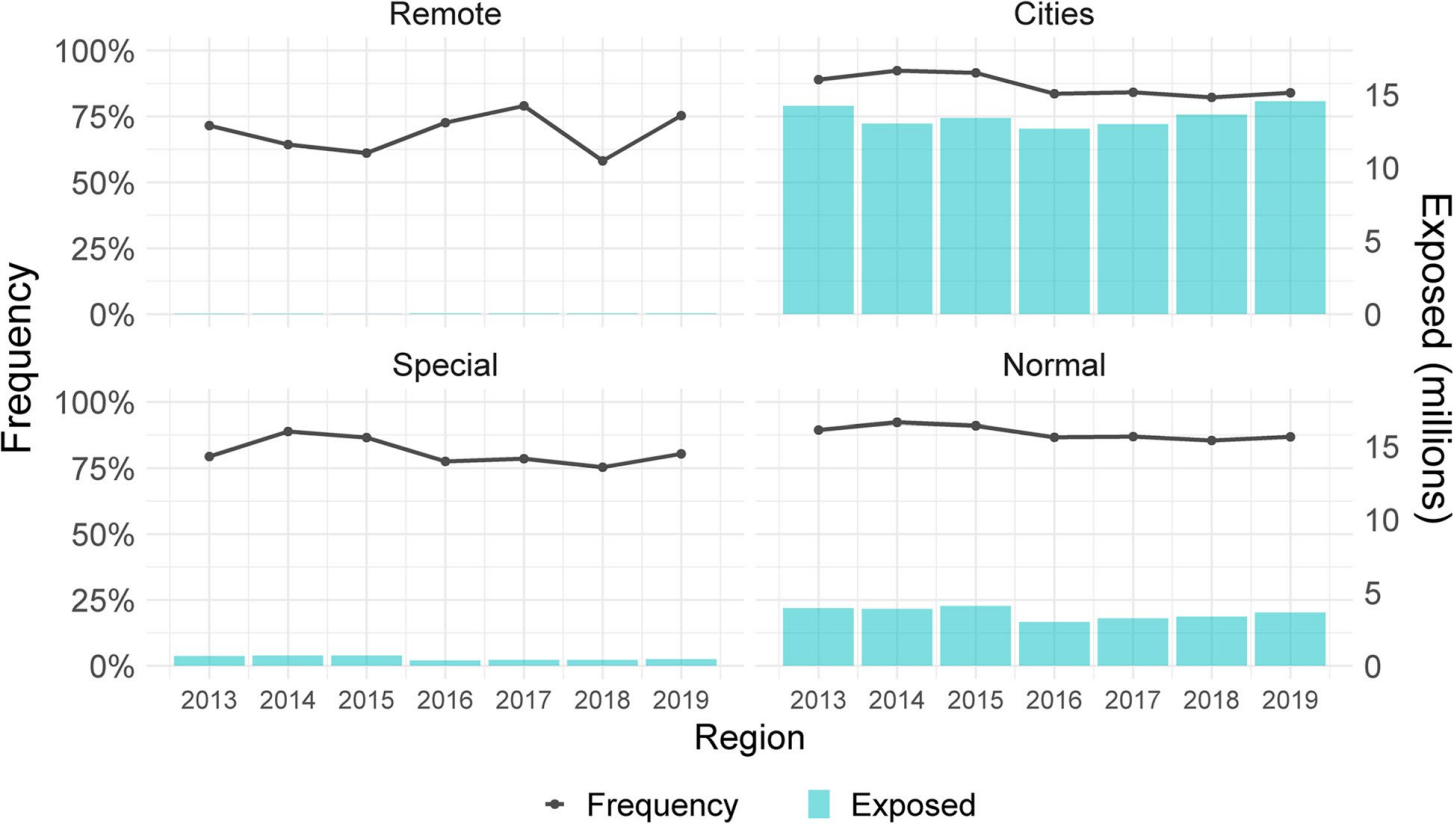
Frequency of people served by the CR by region, 2013–2019

Figure 1 shows the frequency of people served and the number of people exposed of CR by region and year. It can be seen that the frequency is higher in the city and normal regions, and is lower in special and remote regions. The average frequency from 2013 to 2019 was 88.4% in the normal region, followed by cities with 86.7%, special with 81.0% and remote with 68.9%. The ranges for each region over the seven years were: remote (58.1%-79%), cities (82.3%-92.4%), special (75.4%-88.9%), and remote (85.5%-92.4%).

**Fig. A1:**
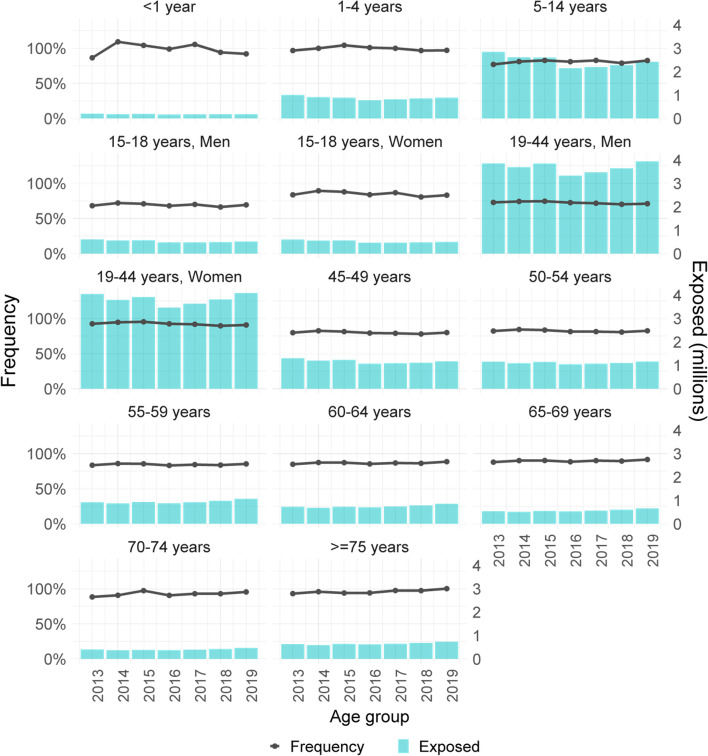
Frequency of people served by the CR by age/sex group, 2013–2019

The observed frequency associated with the age groups is shown in Fig A1 (see Appendix). There are no drastic changes in its evolution over time. On average, the age groups with the highest frequency were in this order: 1 to 4 years, less than one year, 75 years or older, 70 to 74 years, 19 to 44 years (women) and 65 to 69 years, these values are included within the range of 89.5% to 100%. In addition, the age group from 15 to 18 years (men) had the lowest frequency of people attended. On the other hand, the percentage variation of the frequencies between 2013 and 2019 was -0.7% in 15 to 18 years (women), -1.86% in 19–44 years (women) and -2.70% in 19 -44 years (men).

**Fig. 2:**
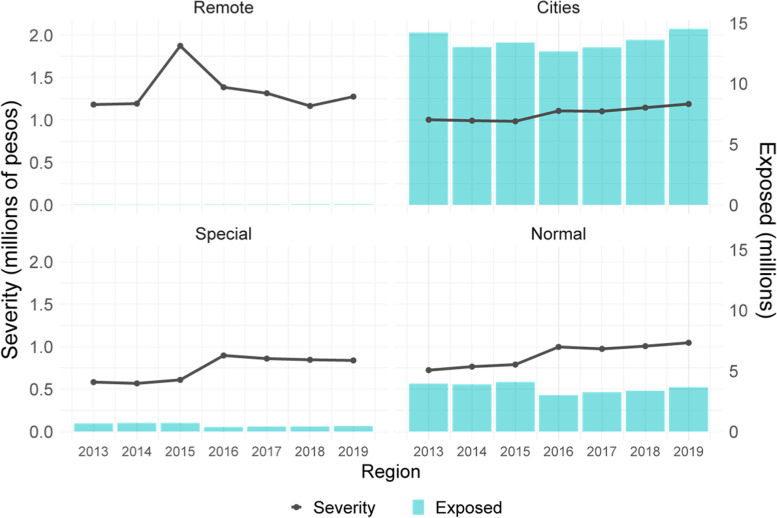
CR severity by region, 2013–2019 (2020 prices)

Figure 2 presents the severity (in 2020 prices, COP) and the number of exposed by region in the CR. From 2013 to 2019, the remote region presents the greatest severity, on average, 1.34 million COP, followed by cities with 1.1 million, normal with 0.9 million and special with 0.7 million. During this period, severity in the remote region grew 7.9% in real terms, in cities 11.5%, in normal region 44.9% and in the special region 43.6%.

**Fig. A2:**
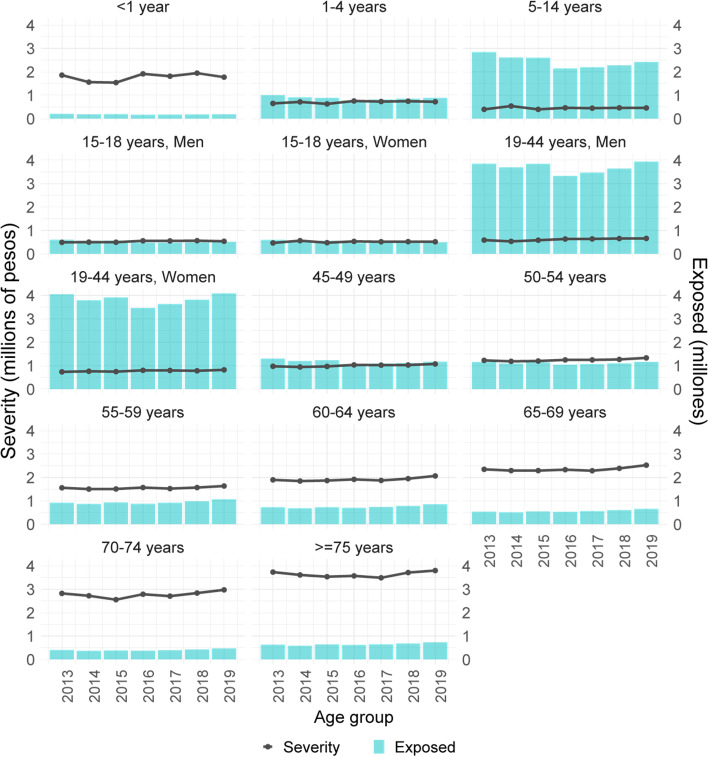
CR severity by age/sex group, 2013–2019 (2020 prices)

With regard to severity by age group, Fig A2 (see Appendix) shows that, from 2013 to 2019, it is greater in groups under one year of age and groups over 60 years of age. Severity maintains a stable value over time for all age groups, except for those under one year of age and those over 70 years of age, where it decreased until 2015 and then increased until 2019. During this time interval, the severity in minors for one year was, on average, 1.8 million COP; in the group from 0 to 4 years, 0.7 million COP; in the groups of men and women from 15 to 18 years and 19 to 44 years it was between 0.4 and 0.8 million COP; in the ages between 45 and 59 years it was around 1.0 and 1.5 million COP; and in groups over 60 years of age it ranged from 2.4 million to 3.8 million COP.

**Fig. 3:**
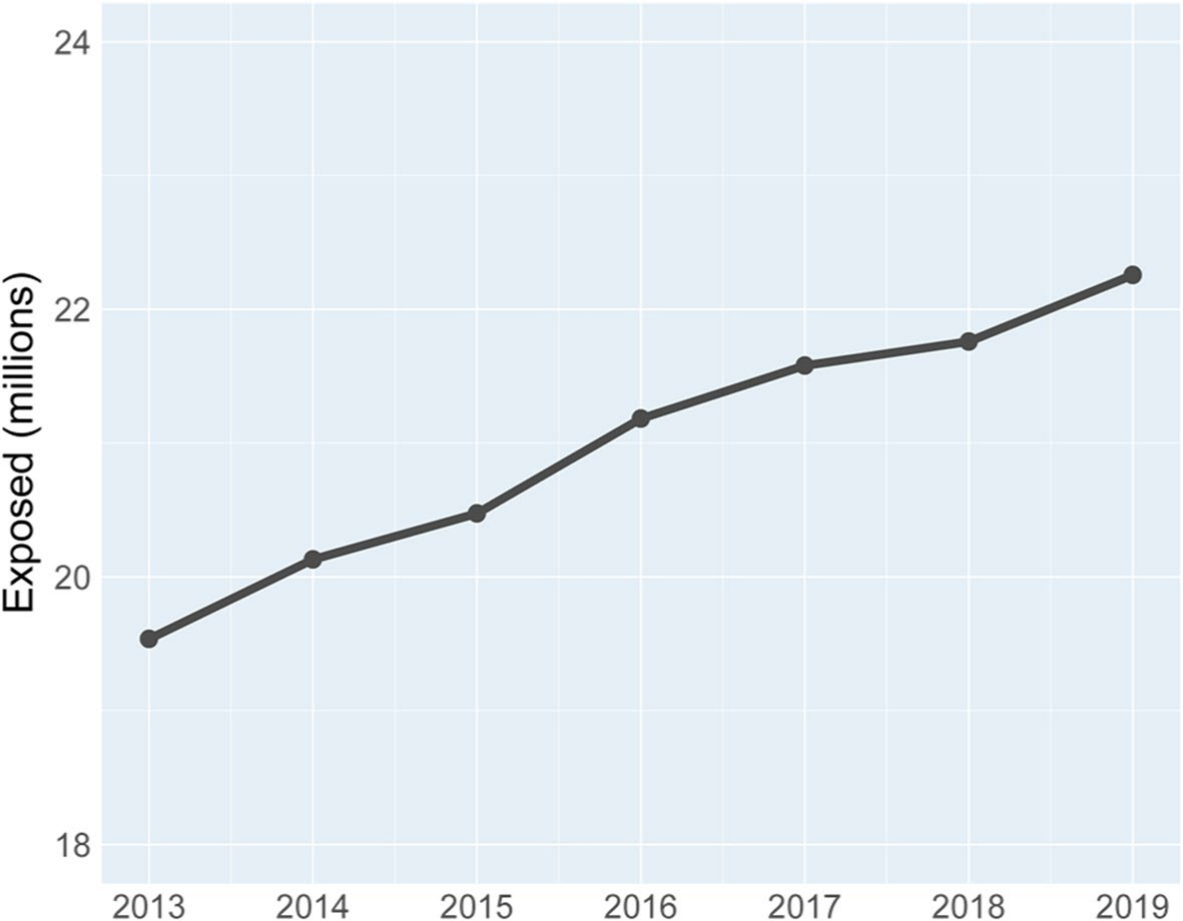
Number of people exposed to CR risk, 2013–2019

Figure 3 shows that the number of people exposed to risk in the CR has grown from 2013 to 2019. In 2013 there were 19.5 million exposed and in 2019 22.3 million, which means a growth of 13.9% over the seven years of analysis.

**Fig. 4:**
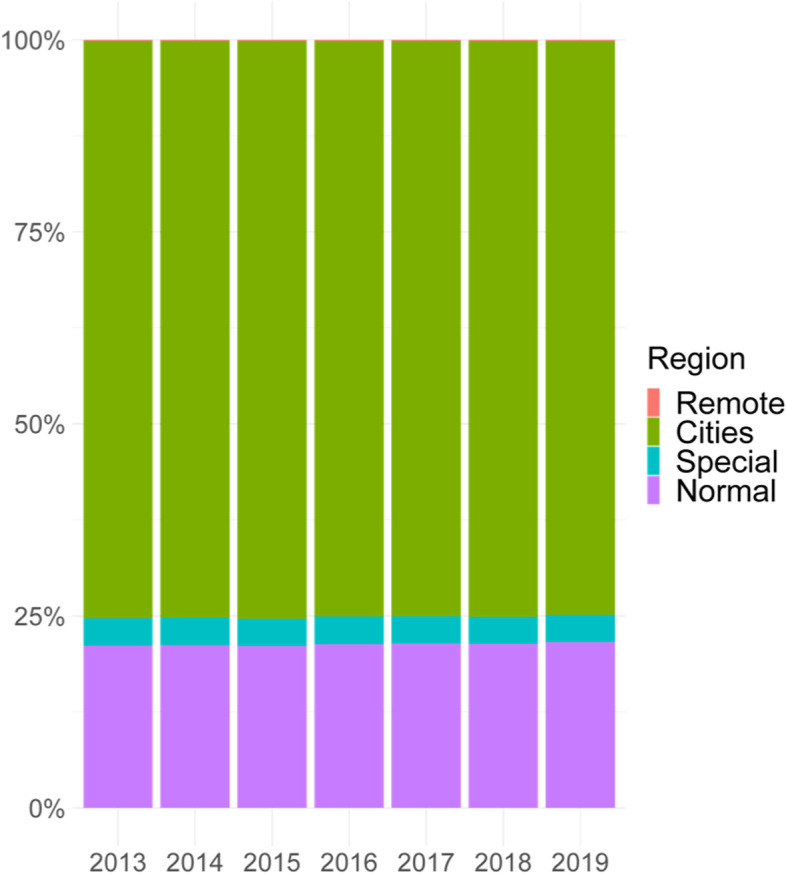
Distribution of those exposed to CR risk by region, 2013–2019

Figure 4 shows the distribution of the number of exposed according to the region between 2013 and 2019. Cities had, on average, 75% of the total exposed, normal 21.2%, special 3.6% and remote only 0.2%. The proportion of those exposed by region was similar throughout the period.

**Fig. A3:**
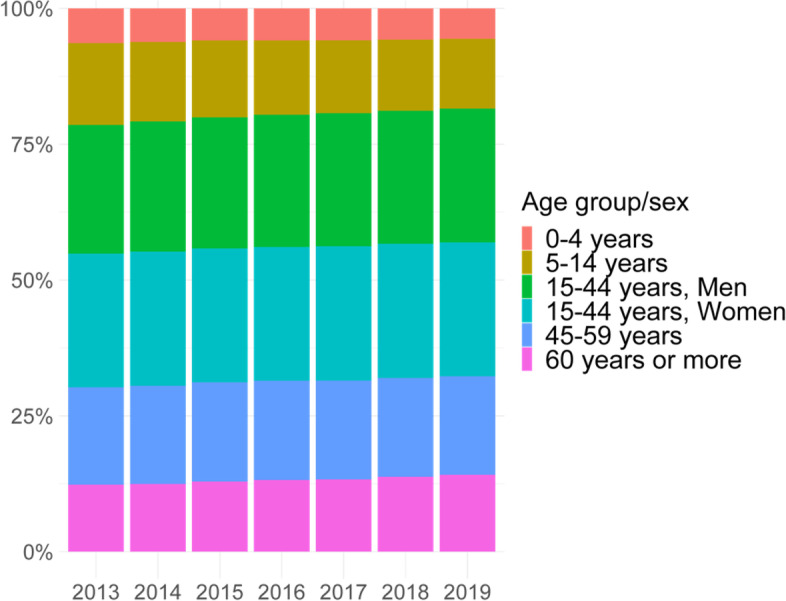
Distribution of those exposed to CR risk by age/sex group, 2013–2019

Finally, Fig A3 (see Appendix) represents the participation of the age groups in the number of exposed to CR risk from 2013 to 2019. On average, the participation in the total number of exposed of the group from 0 to 4 years is 5.9%, of the group from 5 to 14 years is 13.8%, of the men and women from 15 to 44 years is 24.4%, from 45 to 49 years is 18.1% and of the group older than 60 years is 13.2%. The transition towards aging explains the greater growth in the participation of older age groups. The percentage change between 2013 and 2019 in the proportion of those exposed to CR was -12% in the group from 0 to 4 years, -15.2% from 5 to 14 years, 3.8% in men from 15 to 44 years, 0.4% in women from 15 to 44 years, 1.1% from 45 to 59 years and 15.5% in the group over 60 years.

### Empirical strategy

In a first stage, the forecasts of those exposed to the risk are presented, to later detail the process of computation of the adjustment factors for severity and frequency. Afterwards, the explanation of the copula functions and the approach taken for the pricing process of the pure risk premium of the CR is deepened.

#### Forecasts for those at risk

A *deep learning* technique called artificial neural networks (ANN) is used, with high predictive power in demographic, financial and health topics [2, 23, 24, 41, 45, 52]. This type of nonlinear nonparametric model is considered a self-adaptive, accurate method that requires very few assumptions. By simulating the operating system of a biological neuron, ANNs allow for a flexible approach in terms of corresponding functional forms [52]. Thus, the basic architecture of a three-layer fed-forward ANN (one input, one hidden, and one output) is made up of a set of inputs, weights, activation functions, and outputs. Formally:

2 $$\widehat x=\sum_{l=1}^{rs}\varphi_l^2\cdot f\left(\sum_{k=1}^r\varphi_{lk}x_k+\vartheta_{lk}\right)+\vartheta^2,$$

where $$x_k\, (k=1,\dots ,r)$$ is considered the input set, $$\widehat{x}$$ the output set, $$f$$ the activation function, $${\vartheta }_{lk}, {\varphi }_{lk}$$ y $${\varphi }_{j}$$$$(l=1,\dots ,r)$$ the model parameters and weights, and $$rs$$ the neurons in the hidden layers [5]. Then, the ANN training is based on iteratively adjusting these parameters, so that an error function between the forecast $$\widehat{x}$$ and the observation $$x$$ is minimized. This, from the weighted sum of the outputs of the neurons of the hidden layer.

This data science technique is used to forecast, based on historical series, those exposed to risk for the 56 categories defined by the CPU and the adjustment factors.

#### Adjustment factors for severity and frequency

In the statistical-actuarial process of pricing it is necessary to express not only the mean cost of attention per person but also the amount of people in terms of the target year. Likely MSHP this investigation takes evidence of the real world in year $t$ to transform the frequency and severity to $t+2$, so the economic-financial conditions of the health system that are expected in the future for the CR in the country can be represented.

The method for constructing these adjustment factors developed by Basto et al. [3] is closely followed: the following five factors are employed for the severity:

i. Costs incurred but not reported (IBNR): adjust the monetary amount of attention that the EAHBP did not register by the end of the year.
ii. Inclusion of technologies in the HBP-CPU: it recognizes the new basket of sanitary technologies that must be financed in $t+2$, considering the actualization/extension of the HBP-CPU that is made every year.
iii. Comparable: the actual normative is able to finance sanitary technologies not financed by the CPU but are considered as comparable with some of these; in this case the difference between that technology and its comparable is recovered.
iv. Variation in the number of attentions per user: it projects the average number of times that a person receives health technologies.
v. Inflation: it recognizes the rise in the price levels.

For the first three adjustments of severity, the same information from the sufficiency studies of the regulatory entity is used. For the fourth, using GD, the number of monthly attentions per user is forecast, then averaged for the months of year $t+2$. For the fifth factor, forecasts of the *Banco de la República* (the central bank of Colombia) and the Ministry of Finance and Public Credit (MFPC) are taken.

On the other hand, for frequency, two adjustments are considered:

i. Effective coverage advance: recognizes the increment of the rate between users and exposures, this proportion has been increasing in the last years.
ii. Changes in the burden of disease: it adjusts the appearance of new users attended that had not used the health system, due to the occurrence of new health conditions (i.e. new infectious diseases).

The first frequency factor is forecast monthly taking the information of BDUA and GD, then averaging for the months of year $t+2$. In the case of the second factor, a similar quantitative operation is made but taking as a proxy the variable of diagnostics per capita (ICD-10) from GD.

#### Pricing with statistical copulas

In the field of actuarial science, copulas have started to obtain a preponderance at the end of the last century and the first decade of the current century, due to their benefits, in particular the high flexibility of modeling the joint distribution of a random n-tuple [7, 8, 14, 15]. This statistical technique has been applied in several fields of investigation related to the payment of claims, pricing, active valorization and, with less relevance, stockpile computation, highlighting the opportunity to model the asymmetric dependence in the tails [11, 19, 20, 46, 47, 51]. More recently, copulas have been applied in collective risk models and deductible price-fixing, furthermore, improvements in the computational efficiency and how to provide intuitive interpretations of the dependence structure have been investigated [13, 39, 48]. For the sector of health insurance, the applications in the scientific indexed literature have been few [49, 54, 56], and in that way, this work can also be considered a pioneer in the field.

In formal terms, and in a succinct way, a copula is a function that describes the dependence between the marginal probability distributions of two or more random variables and is expressed in terms of a multivariate distribution function. In the bivariate case, let $\left(X,Y\right)$ the random vector with marginal distributions $F\left(x\right)=Pr(X\le x)$ and $G\left(y\right)=Pr(Y\le y)$, respectively, and the joint distribution function $H\left(x,y\right)=Pr(X\le x,Y\le y)$ for $\left(x,y\right)\in {\mathbb{R}}^{2}$ where $F,G,H\sim U\left(\mathrm{0,1}\right)$, the bivariate copula $C$ is a function of the uniform random variables $u=F(x)$ and $v=G(y)$ that are constructed in the following way [18, 38]:

3 $$\begin{array}{c}C:\left[\mathrm{0,1}\right]\times \left[\mathrm{0,1}\right] \to [\mathrm{0,1}]\\ \left(F\left(x\right),G\left(y\right)\right) \mapsto H\left(x,y\right)\end{array} ,$$

and satisfies two properties: i) $\forall u,v\in \left[\mathrm{0,1}\right]$ then $C(u,0)=0=C(0,v)$, $C(u,1)=u$ and $C(1,v)=v$; ii) $\forall {u}_{1},{u}_{2},{v}_{1},{v}_{2}\in \left[\mathrm{0,1}\right]$ with ${u}_{1}\le {u}_{2},{v}_{1}\le {v}_{2}$ then $C\left({u}_{2},{v}_{2}\right)-C\left({u}_{1},{v}_{2}\right)-C\left({u}_{2},{v}_{1}\right)+C\left({u}_{1},{v}_{1}\right)\ge 0$. The first property shows that the contour region of the copula is the consequence of the uniform margin distributions; the second states that $C(u,v)$ is not decreasing in $u$ and $v$. The Sklar theorem (1959) [50] shows that the joint distribution $H$ can be expressed in terms of the marginal distributions $F$ and $G$, and a copula $C$ such that $\forall x,y\in {\mathbb{R}}$:

4 $$\begin{array}{c}H\left(x, y\right)=\mathrm{Pr}\left(X\le x, Y\le y\right)=\mathrm{Pr}\left({F}^{-1}\left(U\right)\le {F}^{-1}\left(u\right), {G}^{-1}\left(V\right)\le {G}^{-1}\left(v\right)\right)\\ =\mathrm{Pr}\left(U\le u, V\le v\right)=C\left(u,v\right)=C\left(F\left(x\right),G\left(y\right)\right),\end{array}$$

where $U=F\left(X\right)$ and $V=G(Y)$ with $U,V\sim U(\mathrm{0,1})$, with $F$ and $G$ as well as their inverse functions monotonic increasing. Moreover, if the marginal distribution functions are continuous, then there exists a unique copula $C\left(F\left(x\right),G\left(y\right)\right)$ equal to $H\left(x,y\right)$. The detailed implications of the different statistical properties can be reviewed in Nelsen [37].

In practice, the most used copula families are Gaussian, *t*-Student, mixed Gaussian and Archimedean. In the last, Gumbel, Clayton and Frank stand out.^5^ The Gaussian and *t*-Student copulas are derived from their own multivariate distributions, for which reason they are called implicit copulas; they also present symmetric dependence but are null or low in the tails [38]. On the other hand, the Archimedean copulas are constructed from a function ${\varphi }_{\theta }:[\mathrm{0,1}]\to [0,\infty ]$ that is continuous, monotone decreasing and convex such that ${\varphi }_{\theta }\left(1\right)=0$, where ${\varphi }_{\theta }$^6^ is referred to as the generator function. Additionally, they describe a great variety of dependence structures, in particular, they allow modeling asymmetric relations between random variables [22, 37].

For the computation of the CR-CPU, defining $X$ as severity (continuous variable) and $Y$ as frequency (discrete variable), it is proposed to model the pure risk premium by a copula, in this case, mixed. The dependence between both variables, following the method developed by Parra [40], includes different covariables through generalized linear models (GLM) in its marginals, which means

5 $${X}_{i}\sim F\left({x}_{i}|{\mu }_{i},\sigma \right); \mathit{ln}\left({\mu }_{i}\right)={\alpha }_{0}+{\alpha }_{l}\sum_{l}Region+{\alpha }_{k}\sum_{k}Age/sex\_group,$$

6 $${Y}_{i}\sim G\left({y}_{i}|{\lambda }_{i}\right);\ \mathit{ln}\left({\lambda }_{i}\right)={\beta }_{0}+{\beta }_{l}\sum_{l}Region+{\beta }_{k}\sum_{k}Age/sex\_group + offset(Exposures).$$

Then, the couple is made by the copula and the joint density function of $X$ and $Y$ is found,

7 $$H\left({x}_{i},{y}_{i}\right)=C\left(F\left({x}_{i}|{\mu }_{i},\sigma \right),G\left({y}_{i}|{\lambda }_{i}\right)\right),$$

8 $$h\left({x}_{i},{y}_{i}|{\upmu }_{i},\sigma ,{\uplambda }_{i}\right)=f\left({x}_{i}|{\upmu }_{i},\sigma \right)*\left[D\left(G\left({y}_{i}|{\uplambda }_{i}\right)|F\left({x}_{i}|{\upmu }_{i},\sigma \right)\right)-D\left(G\left({y}_{i}-1|{\uplambda }_{i}\right)|F\left({x}_{i}|{\upmu }_{i},\sigma \right)\right)\right],$$

where $D\left(v|u\right)$ is the conditional copula of $v$ given $u$ defined as $\frac{\partial C\left(u,v\right)}{\partial u}$.

From Eq. (8) the likelihood is found, and supposing independence between the observations, the parameters of interest of the GLM and the copula are jointly estimated by its maximization with optimization techniques. Once the final parameters are obtained, Monte Carlo techniques are applied to find values for the random variable from samples of the density function. In the present work, 300 samples are simulated (enough to guarantee convergence) and the median is taken as a punctual observation, given its robustness features. Likewise, intervals are constructed from the 2.5 and 97.5 percentiles.

## Results

72 statistical-actuarial models are estimated by year. They come from the combination of the three components, i) severity distributions: Normal, Weibull, Lognormal, Gamma, Inverse Gamma and Inverse Gaussian; ii) frequency distributions: Poisson and Negative Binomial and iii) copula types: two implicit (normal and *t*-Student) and four Archimedean (Clayton, Gumbel, Frank and Joe).

In each year the best model is selected according to the Borda’s rule, which order and rank the 72 rival models according to the values of i) mean square error (MSE); ii) mean absolute percentage error (MAPE); iii) the square root of the square differences between the estimated copula and empirical copula (RSCE) described by Novales [38], and iv) the cross-validation copula information criterion (xvCIC) developed by Grønneberg & Hjort [17]. For each of these criteria, the best model receives 1 point, the second, 2, and so on.

On the other hand, a regularized goodness of fit test is applied for copulas (RGOFC) created by Genest et al. [16] based on a statistic of the Anderson–Darling type,it has a null hypothesis $({H}_{o})$ that the copula presents a good fit. Here a value of one is assigned if at a significance level of 5% the null hypothesis is rejected and zero in the contrary case. Finally, the winning model for each year is the one that has the least total points after summing the points obtained for these five metrics. In Table 1 are shown the results of the five metrics of the chosen models for each year in which the CR-CPU is estimated.

**Table 1:** Evaluation measures of the selected statistical-actuarial models, 2015–2021

CR-CPU actuarial model	2015	2016	2017	2018	2019	2020	2021
Frequency distribution	Negative Binomial	Poisson	Poisson	Negative Binomial	Negative Binomial	Poisson	Poisson
Severity distribution	Weibull	Normal	Normal	Log-normal	Log-normal	Log-normal	Log-normal
Copula type	t-student	Gumbel	Joe	Frank	Frank	Frank	Frank
MSE	2.26E + 10	6.83E + 10	1.82E + 11	2.55E + 09	1.95E + 09	1.36E + 11	2.01E + 10
MAPE	10.7003	11.6370	8.2159	2.9183	2.8590	9.0923	6.5798
RSCE	4.10E-04	4.34E-04	1.97E-04	2.20E-04	5.34E-04	1.96E-04	9.25E-04
xvCIC	2.1730	0.0000	3.94E-08	1.0037	2.5235	2.8837	5.6776
RGOFC	0.9236	0.8566	0.3372	0.6688	0.8357	0.8077	0.8487
Borda rule result	65	75	75	46	58	57	58

**Fig. 5:**
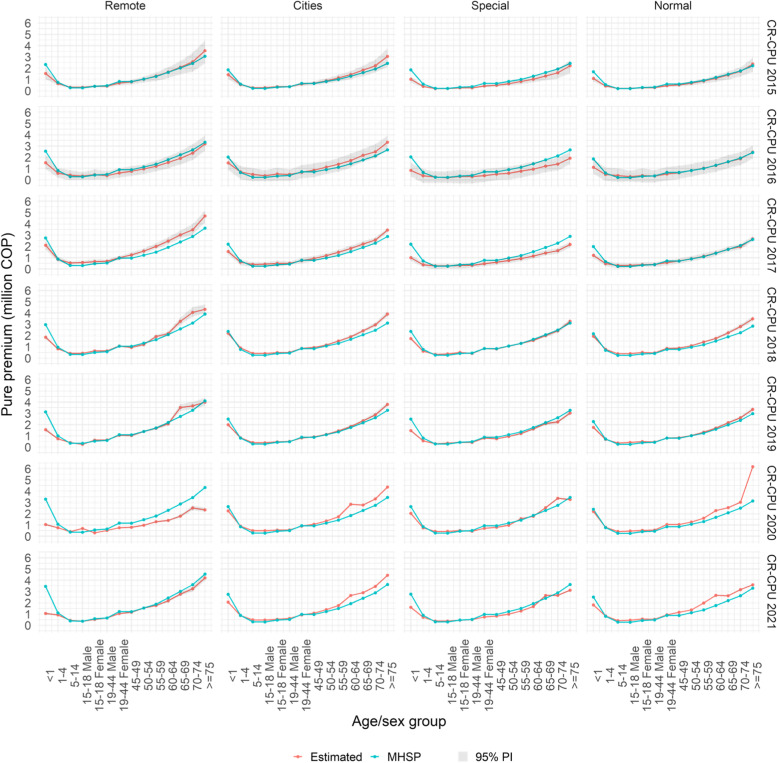
Pure premiums estimated via copulas for the years 2015 to 2021, versus what was calculated by the MSHP

The values in COP, of the pure premium estimated, can be observed graphically in Fig. 5.^7^ There, clear historical patterns are evidenced in relation to the pure premium estimated by MSHP^8^ for each year. In summary, as the first point to stand out, for every region, in every year the pure premium for the group of less than 1 year given by MSHP is higher than that computed in this work.

Second, in the regions ‘cities’ and ‘normal’, in ages 15–18 years (women and men), 65–69 years, 70–74 years and more than 74 years, the pure premium estimated by this study is higher than the one computed by MSHP. As a third point to take into account, for the remote and special regions, the pure premium of MSHP is higher although only slightly than the one estimated by copulas in ages 19–44 years, 45–49 years, 50–54 years. 55–59 years, 60–64 year, 70–74 years and more than 74 years.^9^

## Discussion and conclusions

The present investigation had the objective to estimate actuarially the CR-CPU in the SGSSS of Colombia, in a systematic and strict way, for the period from 2015 to 2021, using modern statistical techniques such as copulas and ANNs. Regarding the sufficiency studies of the CPU developed by the regulatory entity, this work is differentiated in the following topics: i) to compute the pure risk premium, severity and frequency are modeled, then copulas are applied with the purpose of defining the relation of its joint dependence; ii) to forecast the exposures, analytic approximations of deep learning are used, which show benefits over other demographic forecast methodologies; iii) goodness of fit criteria and capacity of forecast are used to select the best estimations and iv) the adjustment factors of Basto et al. [3] for severity and frequency are considered.

For the period 2015–2021, in all regions, the estimated pure premium is very close to the pure premium defined by the MSHP in the age groups 5–14, 15–18 (men and women), 19–44 (men and women). Discrepancy is only observed in the 15–18 group in the remote region in 2017 and 2020 and in the cities region in 2016.

Compared to the authors' estimates, the MHSP underestimated the CPU in age groups 55 years and older in the remote region for the years 2017, 2018 and 2019, in the cities region for the years 2015 to 2021, in the normal region for the years 2018, 2020 and 2021. Instead, the premium is overestimated in age groups over 55 years in the special region for 2016 and 2017. The difference in the estimates for this age group for 2020 are mainly in the remote and normal regions.

Surpluses are observed in the estimated pure premium of the MHSP in the group of less than1 year for the entire period in all regions, mainly remote and special. It is noted that the difference in the estimates for this age group is accentuated with the passing of the years in the remote region.

As a limitation of this study, the approximation here developed is only made for CR, since SR information of spending on health technologies has always had problems of bad quality and little representation, for which reasons there is no data available. It is important to remember that this regime, for 2020, had approximately 23.9 million affiliates and the financing mechanism of the CPU reached values near 24.4 trillion COP [1]. Thence the importance of paying attention to the statistical-actuarial estimations with evidence from the real world.^10^

An adequate estimate of future health spending, as well as the application of efficient risk management mechanisms (from a comprehensive approach) and health technology assessments, will allow better long-term financial sustainability in national public budgets for the health of the population [10, 42]. The methodological development presented here contributes to the international literature in actuarial health sciences, showing innovative analytical developments that may become applicable in other countries with pluralistic health insurance systems. Likewise, this research based on the use of real-world evidence demonstrated the versatility and functionality of statistical copulas (as an inferential modeling technique), which can contribute to informed decision-making in sector financing policy.

Finally, it is important to indicate that this quantitative study is supported and sustained from a prospective approach of computing using the historical data about the spending on health technologies financed with the CR-CPU. Nonetheless, the ideal scenarios for complete effective coverage and integral health services lending (meaning, a CPU from an opportunity/normative approach) is not within the reach of the actual investigation. This last point will require future investigation projects that treat these problems with specificity and the corresponding scenarios. In addition, the authors consider it wise to review in the future the values of the risk weights under a Bayesian approach, which could contribute a certain value-added at the time of adjusting the risk categories, beyond the benefits already explained that result from the use of the statistical copulas presented in this work.

## Data Availability

Data available on request from the authors.

## Appendix

Figs. A1, A2, and A3.

Tables A1, A2, A3, A4, A5, A6, and A7

**Table A1:** Proportion of distinct persons for each reference year

Region	Age/sex group	2013	2014	2015	2016	2017	2018	2019
Remote	Under 1 year	0.6926	0.8258	0.8961	0.9185	0.9138	0.6158	0.7600
Remote	1–4 years	0.9075	0.9309	0.9867	0.9904	1.0000	0.7469	0.9244
Remote	5–14 years	0.6582	0.5887	0.5737	0.6841	0.7292	0.5222	0.7450
Remote	15–18 years, Men	0.5434	0.4374	0.4043	0.5506	0.5773	0.4176	0.5945
Remote	15–18 years, Women	0.6444	0.5554	0.4818	0.6785	0.7145	0.5321	0.7433
Remote	19–44 years, Men	0.5619	0.5076	0.4817	0.5543	0.6370	0.4222	0.5878
Remote	19–44 years, Women	0.8178	0.7424	0.7240	0.8299	0.9041	0.6634	0.8398
Remote	45–49 years	0.7035	0.5942	0.5427	0.6704	0.7120	0.5233	0.6744
Remote	50–54 years	0.7359	0.6058	0.5560	0.7207	0.7617	0.5927	0.7479
Remote	55–59 years	0.7588	0.6778	0.5652	0.7540	0.8203	0.6231	0.7723
Remote	60–64 years	0.7957	0.6919	0.6192	0.7275	0.8478	0.6726	0.7930
Remote	65–69 years	0.7957	0.7137	0.6755	0.8286	0.8950	0.7337	0.8584
Remote	70–74 years	0.8173	0.7486	0.6651	0.8280	0.9029	0.7323	0.8588
Remote	75 years and older	0.8558	0.8000	0.7102	0.8329	0.9168	0.7659	0.9119
Cities	Under 1 year	0.9342	1.0000	1.0000	0.9869	1.0000	0.9391	0.9213
Cities	1–4 years	1.0000	1.0000	1.0000	1.0000	0.9915	0.9600	0.9623
Cities	5–14 years	0.8281	0.8730	0.8800	0.8091	0.8297	0.7909	0.8270
Cities	15–18 years, Men	0.7344	0.7751	0.7532	0.6871	0.7081	0.6673	0.6971
Cities	15–18 years, Women	0.8959	0.9598	0.9342	0.8385	0.8677	0.8019	0.8267
Cities	19–44 years, Men	0.7834	0.7991	0.7946	0.7287	0.7200	0.7069	0.7161
Cities	19–44 years, Women	0.9950	1.0000	1.0000	0.9197	0.9111	0.8935	0.9041
Cities	45–49 years	0.8582	0.8955	0.8701	0.7898	0.7850	0.7775	0.7959
Cities	50–54 years	0.8805	0.9117	0.8921	0.8109	0.8095	0.8043	0.8218
Cities	55–59 years	0.8909	0.9245	0.9098	0.8286	0.8403	0.8350	0.8494
Cities	60–64 years	0.9043	0.9376	0.9264	0.8491	0.8638	0.8580	0.8829
Cities	65–69 years	0.9368	0.9650	0.9571	0.8787	0.8980	0.8911	0.9119
Cities	70–74 years	0.9414	0.9734	0.9718	0.8991	0.9273	0.9253	0.9481
Cities	75 years and older	0.9907	1.0000	0.9961	0.9341	0.9757	0.9700	0.9969
Special	Under 1 year	0.8472	1.0000	1.0000	0.9805	1.0000	0.9278	0.8951
Special	1–4 years	0.9881	1.0000	1.0000	1.0000	1.0000	1.0000	1.0000
Special	5–14 years	0.6938	0.8084	0.7696	0.7092	0.7141	0.6833	0.7444
Special	15–18 years, Men	0.5542	0.6426	0.6132	0.5302	0.5463	0.5285	0.5724
Special	15–18 years, Women	0.7911	0.9097	0.8697	0.7490	0.7848	0.7445	0.7935
Special	19–44 years, Men	0.6686	0.7325	0.7129	0.6360	0.6412	0.6003	0.6352
Special	19–44 years, Women	0.9422	1.0000	1.0000	0.9068	0.9080	0.8671	0.9237
Special	45–49 years	0.7895	0.8609	0.8397	0.7434	0.7748	0.7365	0.7925
Special	50–54 years	0.8140	0.8797	0.8699	0.7723	0.7854	0.7699	0.8260
Special	55–59 years	0.8475	0.9211	0.9187	0.7947	0.8242	0.8108	0.8619
Special	60–64 years	0.8817	0.9700	0.9719	0.8428	0.8642	0.8480	0.9141
Special	65–69 years	0.9307	1.0000	1.0000	0.8603	0.9032	0.8780	0.9468
Special	70–74 years	0.9347	1.0000	1.0000	0.9212	0.9570	0.9270	0.9928
Special	75 years and older	0.9932	1.0000	1.0000	0.9284	0.9661	0.9628	1.0000
Normal	Under 1 year	0.9352	1.0000	1.0000	1.0000	1.0000	0.9802	0.9526
Normal	1–4 years	1.0000	1.0000	1.0000	1.0000	1.0000	1.0000	1.0000
Normal	5–14 years	0.8144	0.8750	0.8406	0.8326	0.8398	0.8105	0.8419
Normal	15–18 years, Men	0.6865	0.7349	0.7046	0.6799	0.6969	0.6767	0.6999
Normal	15–18 years, Women	0.8884	0.9503	0.9075	0.8690	0.8894	0.8484	0.8696
Normal	19–44 years, Men	0.7703	0.7781	0.7710	0.7363	0.7244	0.7119	0.7108
Normal	19–44 years, Women	1.0000	1.0000	1.0000	0.9766	0.9696	0.9484	0.9564
Normal	45–49 years	0.8590	0.8744	0.8579	0.8206	0.8228	0.8126	0.8315
Normal	50–54 years	0.8876	0.8974	0.8874	0.8410	0.8454	0.8384	0.8610
Normal	55–59 years	0.9098	0.9211	0.9160	0.8625	0.8753	0.8749	0.8922
Normal	60–64 years	0.9326	0.9510	0.9404	0.8911	0.9071	0.9083	0.9328
Normal	65–69 years	0.9632	0.9906	0.9785	0.9213	0.9415	0.9400	0.9718
Normal	70–74 years	0.9732	0.9960	1.0000	0.9525	0.9702	0.9807	1.0000
Normal	75 years and older	1.0000	1.0000	1.0000	0.9936	1.0000	1.0000	1.0000

**Table A2:** Values of the frequency adjustment factors for each pricing year

Fit type	2013 $\to$ 2015	2014 $\to$ 2016	2015 $\to$ 2017	2016 $\to$ 2018	2017 $\to$ 2019	2018 $\to$ 2020	2019 $\to$ 2021
Effective coverage	1.23%	5.68%	3.62%	0.96%	1.38%	1.93%	1.27%
Disease burden	0.14%	4.97%	7.82%	1.61%	1.44%	8.27%	5.31%

**Table A3:** Values of the severity adjustment factors for each pricing year

Fit type	2013 $\to$ 2015	2014 $\to$ 2016	2015 $\to$ 2017	2016 $\to$ 2018	2017 $\to$ 2019	2018 $\to$ 2020	2019 $\to$ 2021
IBNR	2.61%	2.71%	2.45%	2.69%	3.11%	3.31%	3.74%
Inclusion of technologies	0%	0.09%	0.03%	0.28%	0.76%	0%	0.65%
Comparable	0.34%	0.27%	0.34%	0.35%	0.34%	0.34%	0.01%
Number of attentions	0.27%	5.58%	2.08%	10.96%	3.16%	10.20%	6.76%
Inflation	5.99%	9.18%	11.82%	7.97%	6.86%	6.73%	4.99%

**Table A4:** Estimated pure premium – CR-CPU for the remote region (COP)

Effective year	2015		2016		2017		2018		2019		2020		2021	
**Age/sex group**	**Copula**	**MHSP**	**Copula**	**MHSP**	**Copula**	**MHSP**	**Copula**	**MHSP**	**Copula**	**MHSP**	**Copula**	**MHSP**	**Copula**	**MHSP**
Under 1 year	1,527,237	2,320,485	1,508,564	2,539,778	2,105,366	2,748,032	1,842,373	2,963,207	1,544,364	3,120,554	1,028,797	3,287,826	1,055,248	3,458,133
1–4 years	634,899	745,113	582,525	815,527	850,385	882,398	810,835	951,494	743,238	1,002,019	747,229	1,055,728	927,376	1,110,416
5–14 years	299,294	260,282	410,646	284,880	526,003	308,237	424,145	332,372	398,523	350,024	405,041	368,787	444,747	387,886
15–18 years, Men	306,240	248,084	330,218	271,528	564,889	293,793	435,342	316,797	246,661	333,620	683,662	351,504	358,514	369,710
15–18 years, Women	390,085	392,024	447,448	429,073	645,779	464,256	631,040	500,606	622,555	527,190	299,330	555,449	520,348	584,221
19–44 years, Men	407,846	441,437	381,247	483,155	688,408	522,774	627,969	563,708	631,857	593,639	518,382	625,463	656,449	657,859
19–44 years, Women	674,254	818,997	611,898	896,398	990,983	969,897	1,059,223	1,045,843	1,051,801	1,101,380	747,168	1,160,416	1,048,619	1,220,524
45–49 years	779,431	810,084	757,177	886,642	1,249,923	959,345	932,069	1,034,461	1,026,460	1,089,392	791,524	1,147,786	1,161,947	1,207,240
50–54 years	1,014,144	1,033,230	956,565	1,130,873	1,582,293	1,223,599	1,190,627	1,319,412	1,386,178	1,389,471	972,547	1,463,952	1,540,702	1,539,784
55–59 years	1,311,325	1,263,017	1,199,510	1,382,375	2,002,300	1,495,727	1,908,277	1,612,846	1,682,974	1,698,489	1,285,237	1,789,533	1,755,720	1,882,229
60–64 years	1,653,070	1,625,489	1,535,564	1,779,100	2,474,964	1,924,981	2,166,413	2,075,712	2,063,106	2,185,934	1,395,306	2,303,105	2,179,174	2,422,405
65–69 years	2,090,396	2,021,971	1,896,163	2,213,053	3,014,590	2,394,516	3,254,871	2,582,011	3,512,683	2,719,115	1,779,592	2,864,870	2,762,948	3,013,268
70–74 years	2,557,457	2,426,349	2,369,414	2,655,647	3,467,850	2,873,400	4,038,312	3,098,393	3,663,509	3,262,920	2,503,696	3,437,821	3,241,089	3,615,898
75 years and older	3,545,571	3,049,021	3,187,219	3,337,164	4,694,169	3,610,802	4,313,430	3,893,534	3,985,410	4,100,282	2,335,281	4,320,067	4,204,190	4,543,847

**Table A5:** Estimated pure premium – CR-CPU for cities region (COP)

Effective year	2015		2016		2017		2018		2019		2020		2021	
**Age/sex group**	**Copula**	**MHSP**	**Copula**	**MHSP**	**Copula**	**MHSP**	**Copula**	**MHSP**	**Copula**	**MHSP**	**Copula**	**MHSP**	**Copula**	**MHSP**
Under 1 year	1,417,639	1,848,647	1,495,897	2,023,351	1,549,862	2,189,255	2,192,416	2,360,680	1,992,175	2,486,033	2,249,336	2,619,291	2,062,813	2,754,962
1–4 years	545,145	593,604	704,723	649,701	605,655	702,976	890,544	758,021	825,014	798,271	896,210	841,062	870,743	884,627
5–14 years	265,372	207,357	476,427	226,952	390,373	245,563	419,110	264,789	400,618	278,851	482,861	293,797	482,894	309,015
15–18 years, Men	273,057	197,640	358,956	216,319	420,098	234,054	404,664	252,383	390,009	265,784	481,441	280,030	464,924	294,535
15–18 years, Women	347,593	312,313	524,442	341,826	483,205	369,856	483,776	398,815	469,554	419,991	541,022	442,506	539,032	465,426
19–44 years, Men	356,061	351,679	474,450	384,912	498,162	416,473	501,367	449,083	497,928	472,933	552,551	498,283	611,970	524,093
19–44 years, Women	594,815	652,468	664,466	714,128	737,620	772,682	850,025	833,185	815,883	877,428	895,888	924,459	925,101	972,347
45–49 years	665,754	645,366	850,475	706,356	916,634	764,274	926,456	824,117	903,457	867,879	1,061,213	914,399	1,087,156	961,765
50–54 years	872,697	823,135	1,117,657	900,924	1,175,257	974,796	1,153,373	1,051,127	1,130,572	1,106,943	1,344,862	1,166,277	1,394,460	1,226,687
55–59 years	1,140,565	1,006,201	1,383,772	1,101,289	1,472,817	1,191,591	1,498,855	1,284,897	1,445,624	1,353,124	1,726,276	1,425,655	1,755,595	1,499,501
60–64 years	1,431,365	1,294,970	1,700,022	1,417,348	1,820,470	1,533,563	1,882,579	1,653,644	1,835,829	1,741,455	2,836,409	1,834,802	2,646,195	1,929,838
65–69 years	1,823,070	1,610,831	2,170,928	1,763,059	2,213,094	1,907,621	2,406,478	2,056,995	2,332,135	2,166,222	2,761,585	2,282,337	2,892,263	2,400,555
70–74 years	2,217,021	1,932,984	2,489,540	2,115,658	2,562,863	2,289,131	2,952,027	2,468,378	2,862,306	2,599,449	3,317,008	2,738,788	3,452,787	2,880,648
75 years and older	3,039,649	2,429,047	3,339,693	2,658,598	3,448,176	2,876,592	3,898,278	3,101,837	3,788,886	3,266,545	4,358,979	3,441,641	4,442,901	3,619,909

**Table A6:** Estimated pure premium – CR-CPU for special region (COP)

Effective year	2015		2016		2017		2018		2019		2020		2021	
**Age/sex group**	**Copula**	**MHSP**	**Copula**	**MHSP**	**Copula**	**MHSP**	**Copula**	**MHSP**	**Copula**	**MHSP**	**Copula**	**MHSP**	**Copula**	**MHSP**
Under 1 year	1,022,841	1,851,002	836,705	2,025,927	992,527	2,192,054	1,719,903	2,363,690	1,471,535	2,489,208	2,026,037	2,622,628	1,600,578	2,758,484
1–4 years	381,148	594,362	361,459	650,530	374,824	703,874	598,096	758,986	556,884	799,292	733,951	842,131	719,895	885,754
5–14 years	186,358	207,622	245,202	227,241	260,301	245,877	332,397	265,126	296,648	279,207	424,887	294,173	383,609	309,410
15–18 years, Men	194,820	197,893	194,575	216,594	263,027	234,352	367,272	252,704	345,051	266,124	430,533	280,386	377,955	294,911
15–18 years, Women	244,472	312,709	291,727	342,261	300,648	370,329	491,309	399,324	431,393	420,529	505,268	443,070	453,328	466,022
19–44 years, Men	252,549	352,126	277,259	385,404	317,296	417,007	409,766	449,657	408,279	473,536	449,851	498,918	503,120	524,760
19–44 years, Women	423,303	653,297	376,866	715,036	459,657	773,670	833,239	834,248	799,255	878,549	695,267	925,639	748,503	973,588
45–49 years	479,420	646,189	509,132	707,256	586,840	765,249	799,963	825,170	734,466	868,987	792,222	915,566	825,343	962,993
50–54 years	619,546	824,185	581,631	902,074	749,439	976,044	1,057,708	1,052,465	944,915	1,108,355	968,409	1,167,764	992,632	1,228,255
55–59 years	819,364	1,007,484	765,185	1,102,692	922,316	1,193,114	1,296,407	1,286,533	1,201,790	1,354,851	1,552,970	1,427,473	1,276,633	1,501,416
60–64 years	1,022,272	1,296,619	935,512	1,419,152	1,141,539	1,535,523	1,553,742	1,655,753	1,608,928	1,743,677	1,765,526	1,837,138	1,665,087	1,932,304
65–69 years	1,298,925	1,612,885	1,209,632	1,765,304	1,408,349	1,910,061	1,986,165	2,059,616	2,095,163	2,168,986	2,526,658	2,285,247	2,650,982	2,403,623
70–74 years	1,594,575	1,935,446	1,390,391	2,118,354	1,621,554	2,292,057	2,415,206	2,471,524	2,238,081	2,602,767	3,372,067	2,742,278	2,660,841	2,884,329
75 years and older	2,214,597	2,432,142	1,912,692	2,661,984	2,166,099	2,880,269	3,264,935	3,105,789	3,022,410	3,270,715	3,268,518	3,446,028	3,113,878	3,624,536

**Table A7:** Estimated pure premium – CR-CPU for normal region (COP)

Effective year	2015		2016		2017		2018		2019		2020		2021	
**Age/sex group**	**Copula**	**MHSP**	**Copula**	**MHSP**	**Copula**	**MHSP**	**Copula**	**MHSP**	**Copula**	**MHSP**	**Copula**	**MHSP**	**Copula**	**MHSP**
Under 1 year	1,089,246	1,682,733	1,110,173	1,841,752	1,202,462	1,992,772	1,925,328	2,148,810	1,760,026	2,262,913	2,199,322	2,384,209	1,816,719	2,507,708
1–4 years	415,654	540,328	481,795	591,391	477,355	639,884	765,132	689,987	684,052	726,628	783,763	765,576	828,879	805,231
5–14 years	200,316	188,746	379,401	206,582	302,489	223,524	384,539	241,024	354,614	253,825	420,214	267,430	422,288	281,281
15–18 years, Men	206,028	179,901	284,127	196,901	316,979	213,049	388,721	229,729	407,991	241,931	457,309	254,897	451,090	268,100
15–18 years, Women	264,823	284,281	379,881	311,147	363,720	336,662	500,229	363,023	487,110	382,297	514,862	402,790	558,194	423,656
19–44 years, Men	271,091	320,115	335,662	350,367	402,197	379,096	461,455	408,781	448,792	430,486	540,138	453,561	535,278	477,054
19–44 years, Women	446,246	593,908	518,118	650,035	561,305	703,336	849,737	758,406	807,323	798,679	1,033,361	841,490	933,274	885,077
45–49 years	515,979	587,444	628,366	642,959	705,847	695,680	872,501	750,154	825,491	789,987	1,032,640	832,333	1,154,243	875,445
50–54 years	663,234	749,260	825,416	820,067	906,885	887,310	1,088,301	956,788	1,022,411	1,007,595	1,250,903	1,061,602	1,366,329	1,116,591
55–59 years	863,344	915,896	1,020,696	1,002,450	1,130,411	1,084,645	1,415,318	1,169,578	1,326,168	1,231,683	1,595,939	1,297,704	1,977,790	1,364,921
60–64 years	1,099,597	1,178,744	1,259,105	1,290,139	1,408,175	1,395,928	1,729,611	1,505,229	1,688,469	1,585,160	2,268,857	1,670,126	2,652,457	1,756,637
65–69 years	1,399,939	1,466,259	1,583,360	1,604,824	1,745,685	1,736,413	2,233,208	1,872,380	2,155,814	1,971,806	2,529,259	2,077,498	2,621,585	2,185,108
70–74 years	1,721,530	1,759,501	1,864,593	1,925,775	1,970,230	2,083,683	2,790,545	2,246,843	2,604,899	2,366,149	3,018,853	2,492,982	3,170,224	2,622,113
75 years and older	2,366,840	2,211,041	2,465,675	2,419,988	2,663,443	2,618,419	3,482,871	2,823,449	3,339,433	2,973,377	6,168,901	3,132,753	3,597,792	3,295,025

.

## Abbreviations

CPU: Capitation Payment Unit
SGSSS: General System of Social Security in Health (*Sistema General de Seguridad Social en Salud*, acronym in Spanish)
MHSP: Ministry of Health and Social Protection
HBP-CPU: Health Benefits Plan
ADRES: SGSSS Resources Administrator (acronym in Spanish)
EAHBP: Entities Administrators of Health Benefit Plans
COP: Colombian pesos
CR: Contributory regime
SR: Subsidized regime
IBNR: Incurred but not reported
DANE: National Administrative Department of Statistics (*Departamento Administrativo Nacional de Estad**í**stica,* acronym in Spanish)
BDUA: Database of Affiliates (*Base de Datos Única de Afiliados*, acronym in Spanish)
GD: Demand Management (*Gestión de la Demanda*, acronym in Spanish)
SISPRO: Integrated Social Protection Information System (acronym in Spanish)
ANN: Artificial neural networks
MFPC: Ministry of Finance and Public Credit
GLM: Generalized linear models
MSE: Mean square error
MAPE: Mean absolute percentage error
RSCE: Square root of the square differences between the estimated copula and empirical copula
xvCIC: Cross-validation copula information criterion
RGOFC: Regularized goodness of fit test for copulas

## Footnotes

1 Of which people who have the ability to pay and contribute jointly and severally to the SGSSS are part (their respective beneficiaries are also included).

2 People who cannot pay their affiliation to the SGSSS (essentially people in conditions of vulnerability and poverty) are welcomed here, being subsidized by the State.

3 Unfortunately for the SR there is no public financial information to calculate its CPU. Historically, the EAHBPs belonging to this regime have had significant quality problems in their administrative records [31, 33].

4 Understanding by someone exposed to risk, an individual who was affiliated with the CR of the SGSSS for a full calendar year.

5 A wide review of other types of copula can be found in Nelsen [37] and Latorre [22].

6 $\theta$ is the parameter of the Arquimedean copula, which is defined in the bivariate case as $C\left(u,v\right)={\varphi }_{\theta }^{-1}\left({\varphi }_{\theta }\left(u\right)+{\varphi }_{\theta }\left(v\right)\right)$.

7 The monetary values of the pure risk premium, the proportion of distinct persons for each reference year and the values of the frequency/severity adjustment factors for each pricing year are presented in Appendix A (Tables A1, A2, A3, A4, A5, A6, A7).

8 The pure premium of the MHSP is found by multiplying the values of the CR-CPU set out in the resolutions of the entity by the percentage spent by the health insurance not allocated to utilities and administration, which is 0.90 in the case of the studied regime [26].

9 The analysis developed here make reference to the punctual estimations of the pure premium for the different categories, not to its confidence intervals.

10 In the year 2021, for the first time in the history of the country, the MHSP used proper information of the SR in the actuarial estimation of the CPU of 2022.
